# Metabolic Syndrome Components and Cancer Risk in Normal-Weight Subjects: Systematic Review and Meta-Analysis in over 18 Million Individuals

**DOI:** 10.3390/jcm15020538

**Published:** 2026-01-09

**Authors:** Yasmin Ezzatvar, Jorge Olivares-Arancibia, Jacqueline Páez-Herrera, Rodrigo Yáñez-Sepúlveda, Óscar Caballero

**Affiliations:** 1 Vicerrectoría de Investigación y Postgrado, Universidad de Los Lagos, Osorno 8050000, Chile; 2 Lifestyle Factors with Impact on Ageing and Overall Health (LAH) Research Group, Department of Nursing, University of València, 46010 Valencia, Spain; 3 Grupo AFySE, Investigación en Actividad Física y Salud Escolar, Escuela de Pedagogía en Educación Física, Facultad de Educación, Universidad de Las Américas, Santiago 7500975, Chile; jolivares@udla.cl; 4 Grupo Investigación Efidac, Escuela Educación Física, Pontificia Universidad Católica de Valparaíso, Valparaíso 2340000, Chile; 5 Faculty Education and Social Sciences, Universidad Andres Bello, Viña del Mar 2521000, Chile; rodrigo.yanez.s@unab.cl; 6 School of Medicine, Universidad Espíritu Santo (UEES), Samborondón 091650, Ecuador; 7 Department of Nursing, University of València, 46010 Valencia, Spain

**Keywords:** metabolic syndrome, hyperglycemia, waist circumference, normal weight, cancer

## Abstract

**Background/objectives:** Metabolic abnormalities, independent of excess weight, may contribute to cancer risk even among individuals of normal weight, though their role remains unclear. This study sought to ascertain if metabolically unhealthy normal-weight (MUNW) individuals, generally characterized by a normal body mass index alongside the presence of metabolic abnormalities, have higher cancer risk than metabolically healthy peers, to analyze variations in risk across obesity-related cancer types, and to examine which single specific metabolic components can predict cancer independently in normal-weight individuals. **Methods:** Two authors systematically searched the PubMed, EMBASE, and Web of Science databases for longitudinal studies, published from inception to July 2025, that included normal-weight adults, classified participants by metabolic health status, and reported incident cancer outcomes in metabolically unhealthy versus healthy normal-weight groups. Hazard ratio (HR) estimates were extracted from each study and were pooled using random-effects inverse-variance model with empirical Bayes variance estimator. **Results:** Thirty-five studies involving 18,210,858 participants (56.0% females, mean age = 53.8 years) were included. A total of 280,828 new cancer cases were diagnosed during follow-up (mean = 10.6 years). In comparison with metabolically healthy normal-weight individuals, MUNW individuals had a 20% higher risk of cancer (HR = 1.20, 95% confidence interval [CI]: 1.13–1.28). Increased risks were observed for gastric cancer (HR = 1.40, 95% CI: 1.04–1.87), pancreatic cancer (HR = 1.37, 95% CI: 1.21–1.54), and colorectal cancer (HR = 1.34, 95% CI: 1.14–1.57), which were the cancer types showing statistically significant associations in subgroup analyses. Normal-weight participants presenting specific metabolic factors like central adiposity or glucose metabolism abnormalities had a 20% (HR = 1.20, 95% CI: 1.13–1.37) and 23% (HR = 1.23, 95% CI: 1.06–1.41) increased cancer risk, respectively. **Conclusions**: MUNW individuals are at higher risk of cancer, with specific metabolic abnormalities, particularly central adiposity and impaired glucose regulation, emerging as the factors most strongly associated with increased risk in normal-weight individuals. Routine metabolic screening and detailed phenotyping are crucial to identify these risks.

## 1. Introduction

Excess body weight is a well-established risk factor for many diseases [1,2], although it is not the sole determinant of disease risk. Most of the global population falls within the normal-weight range [3], yet many individuals in this category may still exhibit some underlying factors that increase susceptibility to chronic diseases, including cancer. While cancer prevention efforts have placed significant emphasis on obesity [4], growing evidence suggests that normal-weight individuals with metabolic dysfunction may also face significant risks [5].

Metabolic abnormalities—such as insulin resistance, dyslipidemia, high blood pressure, central adiposity, and systemic inflammation—are now recognized as silent precursors to disease development and critical determinants of chronic disease risk [6,7,8]. Individuals within the normal-weight range who exhibit at least three of these metabolic syndrome criteria are often categorized as metabolically unhealthy normal-weight (MUNW) and have been shown to face significantly higher risks for major cardiac events and mortality [9]. Recent narrative reviews also suggest that the MUNW phenotype may elevate the risk of developing obesity-related cancers, even in the absence of excess body weight [5,6]. However, these reviews have not quantified this risk nor evaluated whether specific metabolic abnormalities contribute independently to cancer development.

In addition, previous meta-analyses on metabolic dysfunction and cancer risk have not isolated the normal-weight population, making it difficult to determine whether risk patterns differ from those observed in overweight or obese individuals. Likewise, no meta-analyses to date have quantified the cancer risk specifically associated with the MUNW phenotype, where ‘metabolically unhealthy’ refers to the presence of metabolic syndrome, defined by meeting at least three of the established criteria. Moreover, it remains unknown if individual components of metabolic dysfunction—such as elevated waist circumference, triglycerides, fasting blood glucose or blood pressure—are significantly associated with a higher cancer risk in normal-weight individuals. Identifying such markers could help to detect at-risk individuals and enable timely interventions; therefore, this meta-analysis aimed: (1) to ascertain whether MUNW individuals have higher cancer risk than metabolically healthy peers, (2) to analyze variations in risk across obesity-related cancer types, and (3) to examine whether individual metabolic components independently predict cancer in normal-weight individuals.

## 2. Materials and Methods

### 2.1. Ethical Considerations

This study is a meta-analysis of previously published research and does not involve any new data collection from human participants. Therefore, Institutional Review Board approval and informed consent were not required.

### 2.2. Protocol and Registration

The present study was registered in the International Prospective Register of Systematic Reviews (PROSPERO) (Registration number: CRD42024615325) and conducted according to the Preferred Reporting Items for Systematic Review and Meta-analysis (PRISMA) guidelines (PRISMA Checklist in Supplementary Materials). Two researchers (Y.E. and A.G.-H.) independently performed the entire process from the literature selection to data extraction. Disagreements were resolved through consensus.

### 2.3. Eligibility Criteria

Studies were required to meet the following PECOS criteria: (1) participants, normal-weight individuals (i.e., BMI considered normal as per study-specific or regional thresholds), who were initially free of cancer; (2) exposure, studies had to classify participants according to their metabolic health status (metabolically healthy or metabolically unhealthy), confirmed by the presence of at least one of the following metabolic abnormalities: insulin resistance (e.g., measured by HOMA-IR, fasting insulin levels, elevated serum concentrations of c-peptide levels), dyslipidemia (e.g., elevated triglycerides or low high-density lipoprotein cholesterol [HDL-c]), hypertension (e.g., elevated blood pressure), hyperglycemia (e.g., elevated fasting glucose or diagnosed diabetes), central adiposity (e.g., high waist circumference or waist-to-hip ratio); (3) outcome, incidence of cancer, assessed through clinical diagnoses of cancer (confirmed by medical records); and (4) study design, longitudinal studies with at least 1 year of follow-up. Searching was restricted to articles in English and Spanish languages in peer-reviewed journals. A summary of the PECOS eligibility criteria is provided in Table 1.

### 2.4. Information Sources and Search Strategy

Two investigators independently conducted a systematic literature search in the PubMed, EMBASE, and Web of Science databases from their inception to July 2025. The search strategy was audited by a professional librarian and is described in detail in Supplementary Material S1. It incorporated combinations of the following keywords related to metabolic health: ‘metabolic syndrome,’ ‘metabolic abnormalities,’ ‘metabolically unhealthy,’ ‘metabolically obese,’ ‘central adiposity,’ ‘central obesity,’ ‘waist circumference,’ ‘waist-to-hip ratio,’ ‘hyperglycaemia,’ ‘cholesterol,’ ‘triglycerides,’ ‘high blood pressure,’ ‘hypertension,’ ‘metabolically abnormal,’ and ‘insulin resistance’; combined with terms describing normal weight (‘normal-weight,’ ‘body mass index’) and cancer outcomes (‘cancer,’ ‘neoplasm,’ ‘tumor’). The reference lists of related studies were manually reviewed to ensure no eligible studies were overlooked. Any disagreement was resolved by consensus.

### 2.5. Selection Process

After identifying the studies in the three databases, Zotero reference manager (version 5.0, George Mason University, Fairfax, VA, USA) was used to remove duplicate studies.

### 2.6. Data Collection Process

We extracted the following data from each study: (1) study characteristics (first author’s name, publication year, study location, source of information, follow-up duration, sample size and study design); (2) participant information (sex, age, BMI, cancer type); (3) methods of assessment of components of metabolic syndrome; (4) covariates. Efforts were made to retrieve missing data by contacting the corresponding authors of the original studies when it was required, but no replies were provided. To avoid double-counting participants, we screened for overlapping cohorts by comparing study characteristics including cohort name, recruitment period, geographic region, sample size, and participant demographics. When duplicate or overlapping reports from the same cohort were identified, we included only the publication providing the most complete data and longest follow-up, or the analysis most relevant to our study outcomes.

### 2.7. Risk of Bias Assessment

For the risk of bias evaluation, the Joanna Briggs Institute (JBI) Critical Appraisal Tools for cohort studies were used independently by two authors. Disagreements were resolved by consensus. This tool evaluates risk of bias across 11 items, each with four response options: yes, no, unclear, or not applicable. The total score serves as the overall assessment.

### 2.8. Data Synthesis and Analysis

Associations between metabolic risk factors and cancer incidence were summarized as HRs with 95% CIs. HRs were log-transformed for analysis, and pooled estimates were calculated using a random-effects model based on the Empirical Bayes method [10], comparing MUNW individuals to metabolically healthy normal-weight participants as a reference group in relation to cancer incidence. For clarity, ‘metabolically unhealthy’ was defined as per the criteria used by each included study (i.e., study-specific definitions, which varied and, in some cases, required ≥1 or ≥2 abnormalities, while others used the standard metabolic syndrome definition of ≥3 abnormalities). We therefore performed two pre-specified analyses: (1) a pooled analysis using each study’s author-defined (‘study-specific’) definition of metabolic unhealthiness, which intended to reflect the heterogeneity that exists in currently published research and (2) a restricted analysis including only studies that applied the standard metabolic syndrome criterion (≥3 components), which provides the more clinically meaningful and actionable estimate.

Some considerations were taken in our meta-analysis. First, meta-analyses were conducted only for outcomes that were included in at least three studies. Second, when multiple studies reported data from the same cohort, the study with the largest sample size was selected. Third, when studies reported HRs using different cutoff points, we combined the log-transformed HRs across these cutoff points into a single estimate using a fixed-effect model. Finally, when multiple statistical risk-adjustment models were available, we extracted the HRs from the model with the most extensive covariate adjustment.

The heterogeneity of the results across the studies was estimated by the Q-I2 statistic, where I^2^ values of 25%, 50%, and 75% were used as thresholds to indicate low, moderate, and high heterogeneity, respectively [11]. Subgroup analyses were conducted to explore potential sources of heterogeneity. These analyses focused on specific cancer types or specific components of metabolic syndrome to assess their distinct contributions to the observed effects. All analyses were conducted using Stata version 17 (StataCorp, College Station, TX, USA). All statistical tests were two-sided, and *p* values < 0.05 were considered statistically significant.

### 2.9. Additional Analyses

To test the stability of the results, a sensitivity analysis was performed using the leave-one-out method, which involves systematically removing one study at a time to see if the overall findings remain consistent. Additionally, meta-regressions were performed to examine whether factors such as age or follow-up duration influenced the effect sizes. These meta-regressions were conducted only when data were available from at least five studies. Lastly, small-study effects were evaluated using Doi plots and the Luis Furuya Kanamori (LFK) index, which demonstrate superior sensitivity and diagnostic accuracy compared with traditional funnel plots and Egger’s regression, especially in meta-analyses with a small number of studies. An LFK index value > 1 or falling < −1 indicates minor asymmetry, while values > 2 or falling < −2 suggest major asymmetry [12].

## 3. Results

### 3.1. Study Selection

**Figure 1:**
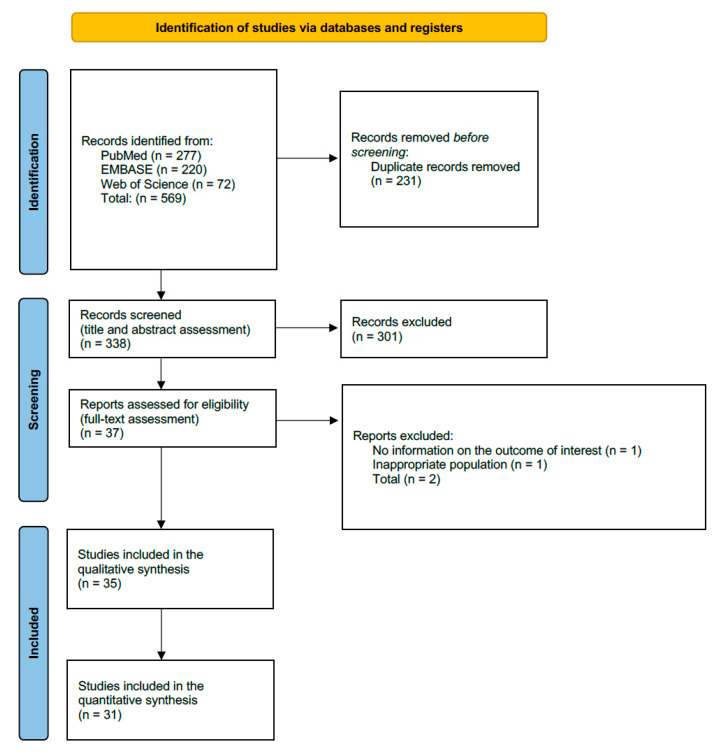
PRISMA flow diagram of literature search and study selection.

The electronic search retrieved 569 articles. After removing duplicates, assessing titles and abstracts and reading full-text, 35 studies were finally included in the systematic review [7,8,13,14,15,16,17,18,19,20,21,22,23,24,25,26,27,28,29,30,31,32,33,34,35,36,37,38,39,40,41,42,43,44,45], and of these, 31 were analyzed in the meta-analysis. A PRISMA flow diagram illustrating the number of studies at each stage of the study is shown in Figure 1. The reference list of excluded studies and reasons for exclusion are provided in Supplementary Material S2.

### 3.2. Study Characteristics

The general characteristics of the 35 included studies are shown in Table 2. The number of participants analyzed was 18,210,858 participants (56.0% females, mean age = 53.8 years). Study designs included prospective cohort and case–control studies, with a mean follow-up of 10.6 years (ranging between 3.7 [25] and 30 [7] years). A total of 280,828 new cases of cancer were diagnosed during the respective study periods. The studies took place in different countries, including China, Canada, United States, South Korea, Japan, Taiwan, Nigeria, Sweden, England, Wales, Scotland, Norway, Austria, Denmark, Italy, the Netherlands, Spain, France, Germany, and Greece.

Most studies defined normal weight as a BMI < 25 kg/m^2^, with some exceptions where cut-offs were BMI < 28 kg/m^2^ [8], BMI < 22.9 kg/m^2^ [22], and BMI < 23.9 kg/m^2^ [28,33]. The definition of metabolic abnormality differed across studies. Most studies [7,8,14,16,18,21,24,29,31,32,35,36,38,40,41,43] followed the metabolic syndrome criteria defined by the National Cholesterol Education Program’s Adult Treatment Panel III, which is based on the presence of three or more criteria of the following: waist circumference ≥ 90 cm, serum triglyceride ≥ 150 mg/dL, HDL-cholesterol < 40 mg/dL, blood pressure ≥ 130/85 mmHg or taking antihypertensive medication, and fasting blood glucose ≥ 100 mg/dL or taking antidiabetic medication. Other studies defined metabolically healthy status as the top tertile of the metabolic score (which comprised blood pressure, plasma glucose and triglycerides) [30], the presence of one or more criteria of metabolic syndrome [13,15,17,19,20,22,23,25,26,27,28,34,37,42], two or more criteria [44,45] or four or more [39] including measures of central adiposity (i.e., waist circumference, waist-to-hip ratio), measures of abnormal glucose metabolism (i.e., fasting blood glucose, insulin levels, C-peptide concentration), triglycerides, hypertension and total cholesterol.

### 3.3. Risk of Bias in Studies

The results of the JBI Appraisal Checklist for Cohort Studies are detailed in Supplementary Material S3. The included studies performed well on most of the 11 items, with a mean score of 9.8, indicating generally strong methodological quality across the evidence base. However, some studies did not fulfill specific checklist items, such as failing to adequately identify confounding factors, and in case–control studies, the criterion requiring the two groups to be similar and recruited from the same population was not met.

### 3.4. Synthesis of Results and Additional Analysis

#### 3.4.1. Cancer Risk in Normal-Weight Individuals with Metabolic Syndrome (Using Any Definition)

**Figure 2:**
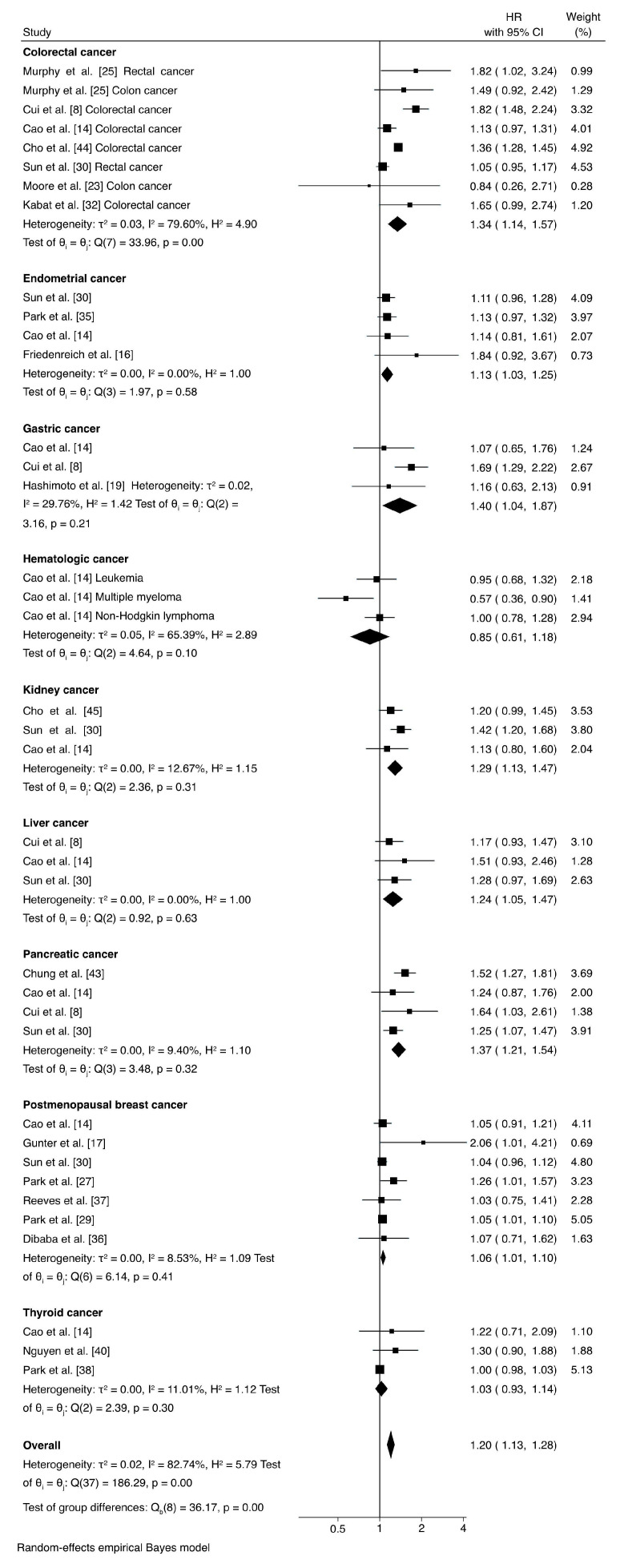
Forest plot showing hazard ratios (HRs) for cancer risk in normal-weight individuals with metabolic syndrome (using any definition) versus normal-weight individuals without metabolic syndrome.

Figure 2 presents the study-level findings by cancer type and the overall pooled results comparing MUNW individuals to those metabolically healthy normal-weight individuals in relation to cancer risk. In this analysis, metabolic syndrome was defined based on the criteria used in each individual study, which varied—some studies required at least three criteria, while others defined metabolic syndrome using only one criterion. Individuals with metabolic syndrome exhibited a statistically significant increased risk for several specific cancers, including gastric (HR = 1.40, 95% CI: 1.04 to 1.87), pancreatic (HR = 1.37, 95% CI: 1.21 to 1.54), colorectal (HR = 1.34, 95% CI: 1.14 to 1.57), kidney (HR = 1.29, 95% CI: 1.13 to 1.47), liver (HR = 1.24, 95% CI: 1.05 to 1.47), endometrial (HR = 1.13, 95% CI: 1.03 to 1.25), or postmenopausal breast cancer (HR = 1.06, 95% CI: 1.01 to 1.10). In contrast, no statistically significant associations were observed for other cancer subtypes, such as hematological (HR = 0.85, 95% CI: 0.61 to 1.18) or thyroid cancer (HR = 1.03, 95% CI: 0.93 to 1.14). Regardless of cancer subtype, our findings indicate an overall 20% increased relative risk of cancer among MUNW individuals based on any definition (HR = 1.20, 95% CI: 1.13 to 1.28). Subgroup analyses by cancer type reduced the heterogeneity to minimal levels, indicating that cancer type was likely the primary source of variability.

#### 3.4.2. Cancer Risk in Normal-Weight Individuals with Metabolic Syndrome (Defined by 3 or More Criteria)

**Figure 3:**
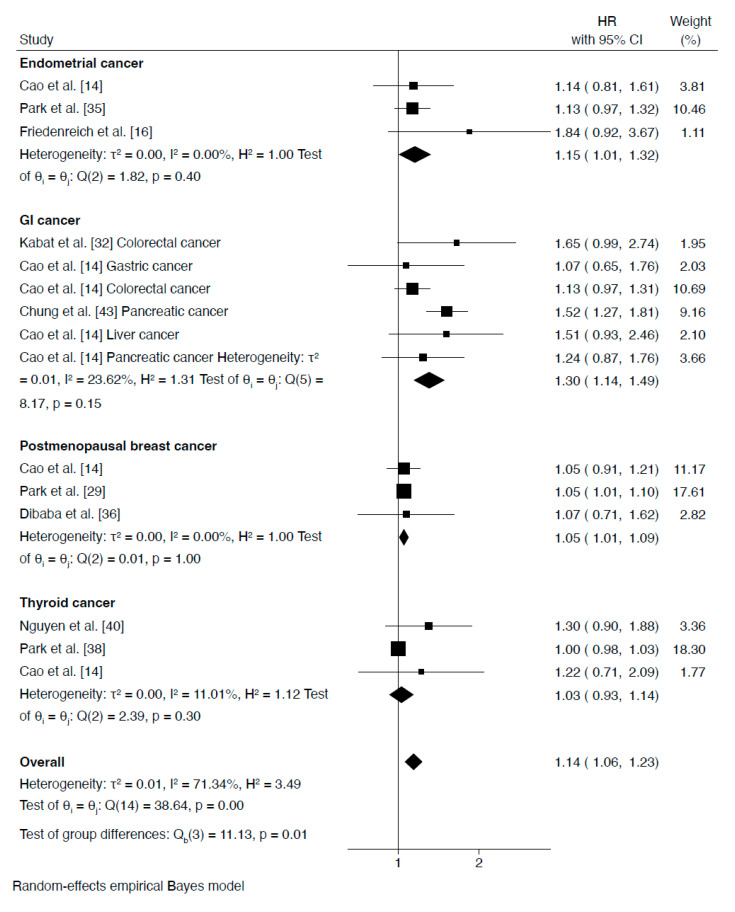
Forest plot showing hazard ratios (HRs) for cancer risk in normal-weight individuals with metabolic syndrome (defined by 3 or more criteria) versus normal-weight individuals without metabolic syndrome.

As shown in Figure 3, when metabolic syndrome was defined as meeting three or more criteria, the risk of developing gastrointestinal cancer was significantly increased (HR = 1.30, 95% CI: 1.14 to 1.49), as well as the relative risk of endometrial cancer (HR = 1.15, 95% CI: 1.01 to 1.32) and postmenopausal breast cancer (HR = 1.05, 95% CI: 1.01 to 1.09). In contrast, no significant association was observed for thyroid cancer (HR = 1.03, 95% CI: 0.93 to 1.14). Low heterogeneity among studies indicated minimal variation in effect sizes across the included analyses.

#### 3.4.3. Cancer Risk Associated with Single Metabolic Components in Normal-Weight Participants

The results showing the association between single specific individual components of metabolic syndrome and cancer risk in normal-weight participants are presented in Supplementary Material S4–S7. For central adiposity (Supplementary Material S4), a high waist circumference was associated with a 19% increased relative risk of developing cancer (HR = 1.19, 95% CI: 1.07 to 1.34), while a high waist-to-hip ratio was linked to a 20% higher cancer risk (HR = 1.20, 95% CI: 1.08 to 1.32). Overall, normal-weight participants with central adiposity had a 19% higher risk of cancer (HR = 1.19, 95% CI: 1.10 to 1.32).

Among normal-weight individuals with glucose metabolism abnormalities (Supplementary Material S5), cancer risk was 23% higher (HR = 1.23, 95% CI: 1.06 to 1.41) compared to metabolically normal peers. Similarly, as shown in Supplementary Material S6, a significant association was observed for normal-weight individuals with high levels of cholesterol (HR = 1.07, 95% CI: 1.02 to 1.13). In contrast, no statistically significant associations were found independently for hypertension (HR = 1.12, 95% CI: 0.99 to 1.26) or elevated triglycerides (HR = 1.06, 95% CI: 0.99 to 1.14) (Supplementary Material S7).

#### 3.4.4. Additional Analyses

We performed a meta-regression analysis to examine whether baseline age or years of follow-up influence the effect size observed across studies (Supplementary Material S8). The results showed a statistically significant positive association between age and the relative risk of cancer (coefficient = 0.022, standard error = 0.011, z = 2.07, *p* = 0.039, 95% CI: 0.001 to 0.044), indicating that, for every one-year increase in the mean age of MUNW individuals, the HR increases by 0.022 units. In contrast, follow-up was not significantly associated with the effect size (coefficient = −0.034, standard error = 0.025, z = −1.36, *p* = 0.173, 95% CI: −0.082 to 0.015).

Sensitivity analysis using the leave-one-out method was carried out to evaluate the influence of each individual study on the overall pooled estimate. By removing one study at a time, we found that the pooled HR remained consistent, indicating that no single study disproportionately influenced the overall results.

Publication bias and small study effects were assessed through Doi plots and LFK index. The results showed significant asymmetry across all assessed variables, suggesting the presence of potential publication bias (Supplementary Material S9).

## 4. Discussion

In this study, we examined the risk for having cancer in MUNW individuals, finding that this phenotype is associated with a 20% higher overall cancer risk compared to metabolically healthy normal-weight individuals, with the highest risks observed for gastrointestinal cancer, endometrial cancer, and postmenopausal breast cancer. Additionally, metabolic syndrome components such as glucose abnormalities, central adiposity, and cholesterol levels were found to be clear independent key metabolic abnormalities in normal-weight individuals. These findings emphasize that metabolic abnormalities may be associated with higher cancer risk, highlighting the need for early detection and targeted interventions in normal-weight individuals.

Our findings are consistent with previous reviews suggesting that metabolic abnormalities are linked to higher cancer risk [5,6]. However, these narrative reviews often applied inconsistent definitions of metabolic dysfunction and classified individuals as ‘metabolically unhealthy,’ even when some studies included participants with a single metabolic abnormality. Our results in MUNW individuals further support this association, suggesting that metabolic dysfunction may act as a precursor of cancer risk. Interestingly, our findings agree with those of a recent meta-analysis comparing metabolically healthy obesity to metabolically unhealthy individuals with obesity, which reported a significantly lower cancer risk in metabolically healthy obese individuals [46], reinforcing the idea that metabolic dysfunction may be a stronger determinant of cancer risk than adiposity alone.

We found that glucose abnormalities, central adiposity, and cholesterol imbalances were independently associated with an increased cancer risk. Our findings suggest that the presence of some of these factors—even before a formal diagnosis of metabolic syndrome—can influence cancer risk, serving as clear key metabolic abnormalities for cancer in normal-weight individuals. Regarding central adiposity, both a high waist circumference and high waist-to-hip ratio were associated with a higher risk of cancer. This is likely due to the metabolic activity of visceral fat, which promotes chronic low-grade inflammation through increased secretion of inflammatory cytokines and adipokines and can alter sex hormone metabolism by converting androgens into estrogen, contributing particularly to hormone-sensitive cancers [47,48,49].

Excess abdominal fat also contributes to insulin resistance, elevating insulin levels, which stimulate cell proliferation and activate the insulin-like growth factor system, promoting cancer cell growth [47]. Furthermore, abnormalities in glucose metabolism alter energy production, as cancer cells shift from oxidative phosphorylation to glycolysis (the Warburg effect), providing a growth advantage [50]. Elevated blood glucose also leads to advanced glycation end-products, which bind to cell receptors, triggering inflammation and tissue damage that contribute to cancer progression [48,50].

Cancer risk was particularly pronounced in specific cancers, such as gastrointestinal, endometrial, and, to a lesser extent, postmenopausal breast cancer, sites known to be particularly sensitive to metabolic disturbances. Gastrointestinal cancers, including colorectal and pancreatic, are strongly associated with metabolic abnormalities, possibly due to their sensitivity to factors like insulin resistance and chronic inflammation [51,52]. Endometrial cancer, on the other hand, is particularly influenced by insulin resistance and the resulting hormonal imbalances, such as increased estrogen levels [53]. Similarly, high visceral fat in postmenopausal women is associated with increased estrogen production compared to leaner women, a well-known contributor to breast cancer development [54].

### 4.1. Clinical Applications

Normal-weight individuals with metabolic abnormalities should be assessed using practical, routinely available screening tools, such as fasting plasma glucose or HbA1c for impaired glucose regulation; lipid profile and blood pressure for cardiometabolic risk; or simple anthropometric measures including waist circumference or waist-to-hip ratio to screen for abdominal obesity, since early detection of these conditions enables timely interventions, potentially preventing the development of full metabolic syndrome and its associated cancer risks. In clinical settings and trials, incorporating these accessible biomarkers into routine risk assessment is recommended to accurately identify metabolic risks and distinguish between metabolically healthy and unhealthy normal-weight individuals, beyond BMI alone. This will help target interventions for individuals at risk of cancer, despite having a normal BMI, and enable tailored lifestyle and pharmacological treatments. Ultimately, this can lead to personalized medicine that addresses the unique needs of each patient.

### 4.2. Strengths and Limitations

It is important to acknowledge some limitations that may influence the interpretation of findings. First, metabolic risk factors were predominantly measured only at baseline, without accounting for potential variations in metabolic changes or BMI fluctuations over time. Thus, potential transitions between healthy and unhealthy states over follow-up may have introduced non-differential misclassification, likely attenuating effect estimates. Second, the studies lacked a universal definition of metabolic health or BMI normal-weight criteria. Although most studies adhered to generally accepted cut-offs and definitions, this variability may have introduced some heterogeneity, emphasizing the need for future research to adopt standardized and validated metabolic health parameters. Third, while many studies adjusted for key covariates such as age, sex, physical activity, income, smoking history, and alcohol consumption—well-recognized cancer risk factors—other critical confounders were often neglected. Specifically, dietary patterns, family history of cancer, and history of infections such as Helicobacter pylori or Clonorchis sinensis, which are well-established contributors to cancer risk, were rarely considered. Unmeasured lifestyle factors (e.g., sleep patterns, environmental exposures) may also contribute to residual confounding, which may bias associations upward or produce spurious links. Additionally, the significant asymmetry detected in the Doi plots and LFK index suggests the presence of publication bias, which may further influence the magnitude of pooled estimates. As a result, the observed effect estimates should be interpreted with caution, and future studies incorporating more complete confounder assessment are needed to better isolate the independent contribution of metabolic abnormalities.

## 5. Conclusions

MUNW individuals appear to be at higher relative risk of developing obesity-related cancers in general, specifically colorectal, breast and gastrointestinal cancers, compared to those metabolically healthy. Central adiposity, glucose metabolism abnormalities or high cholesterol levels were each associated with higher cancer risk in normal-weight individuals, independent of BMI. These observation findings highlight the need to broaden cancer prevention strategies beyond weight reduction to include metabolic health as a key focus, particularly for individuals with normal BMI but underlying metabolic dysfunction. Routine metabolic screening in the normal-weight population is strongly recommended to identify obesity-related metabolic phenotypes early, thereby reducing future cancer risk. Furthermore, future research should explore potential differences in these associations across sex and ethnicity and should include longitudinal studies with repeated metabolic measurements, as well as intervention trials where feasible, to better establish causality and inform targeted prevention strategies. Additionally, future studies using standardized and dynamic metabolic phenotyping and rigorous adjustment for key confounders are needed to clarify the causal relevance and refine prevention strategies across diverse populations.

## Figures and Tables

**Table 1:** PECOS Framework Criteria for Study Eligibility.

	Criteria
Participants	Normal-weight individuals (BMI within normal range per study-specific or regional thresholds) who are free of cancer at baseline.
Exposure	Metabolically unhealthy status, defined as the presence of at least one metabolic abnormality, such as insulin resistance, dyslipidemia, hypertension, hyperglycemia, or central adiposity.
Comparator	Metabolically healthy individuals (absence of metabolic abnormalities).
Outcome	Incident cancer, determined via clinical diagnosis and confirmed through medical records.
Study Design	Longitudinal studies with ≥1 year of follow-up.

**Table 2:** Main characteristics of the included studies.

Study (Year)	Country	Follow-Up (Years)	n (Females)	Age	Definition of Normal Weight	Definition of Metabolically Unhealthy	Type of Cancer	Confounders
Ärnlöv et al. [7]	Sweden	30	955 (0)	49.6	BMI < 25 kg/m^2^	Metabolic syndrome present if 3 or more of the following criteria are fulfilled: FBG ≥ 5.6 mmol/L (100 mg/dL) BP ≥ 130/85 mm Hg or treatment TG ≥ 1.7 mmol/L (150 mg/dL) HDL-c < 1.04 mmol/L (40 mg/dL) BMI ≥ 29.4 kg/m^2^	Overall cancer	Age, smoking, and LDL-c
Arthur et al. [13]	England, Wales and Scotland	7	149,928 (149,928)	53	BMI = 18.5–24.9 kg/m^2^	Body fat measures using BIA (Quintiles): WC, WHR, trunk fat mass index, fat mass index	Postmenopausal breast, endometrium, ovary and colon/rectum	Age at enrollment, education, age at menarche, age at first full-term birth and parity combined, HRT status, age at menopause, height, physical activity, alcohol intake, smoking
Cao et al. [14]	England, Wales and Scotland	7.8	126,857 (81,158)	56.3	BMI = 18.5–24.9 kg/m^2^	MU if ≥2 of the following criteria is fulfilled, otherwise MH: (1) elevated BP, defined as a systolic BP ≥ 130 and/or a diastolic BP ≥ 85 mmHg and/or the use of antihypertensive medication at baseline, (2) hypertriglyceridemia, defined as TG ≥ 1.7 mmol/L (150 mg/dL) or current use of lipid-lowering medication at baseline, (3) low HDL-c, defined as <1.0 mmol/L (40 mg/dL) for men and <1.3 mmol/L (50 mg/dL) for women, (4) hyperglycaemia, defined as FBG ≥ 5.6 mmol/L or use of medications for diabetes at baseline (e.g., insulin or oral antidiabetic medications)	Oral, oesophagus, stomach, colorectal, liver, gallbladder, pancreas, lung, malignant melanoma, postmenopausal breast, cervix, endometrium, ovary, prostate, kidney, bladder, brain and thyroid cancers, and non-Hodgkin lymphoma, multiple myeloma and leukaemia	Sex, age, education attainment, employment, ethnicity, Townsend deprivation index, alcohol intake and smoking status. Models for cervix, ovary and endometrium cancers are additionally adjusted for HRT use, oral contraceptive use and menopause after excluding females with history of hysterectomy. Models for postmenopausal breast cancer were additionally adjusted for HRT and oral contraceptive use
Cho et al. [44]	South Korea	2	204,602 (100,036)	58.8	BMI < 25 kg/m^2^	Metabolic health was defined as having none or one of the following risk factors: (1) systolic BP ≥ 130 mmHg and/or diastolic BP ≥ 85 mmHg and/or taking antihypertensive medications, (2) TG level ≥ 150 mg/dL and/or taking lipid-lowering medications, (3) FPG level ≥ 100 mg/dL and/or taking antidiabetic medications, (4) HDL-c levels < 40 mg/dL in men and <50 mg/dL in women.	Colorectal	Age, sex, income, smoking, alcohol drinking, and presence of inflammatory bowel disease
Cho et al. [45]	South Korea	5.4	205,093 (97,182)	60	BMI < 25 kg/m^2^	Having two or more of the following risk factors: (1) systolic BP ≥ 130 mmHg and/or diastolic BP ≥ 85 mmHg and/or taking antihypertensive medications; (2) TG level ≥ 150 mg/dL and/or taking lipid-lowering medications; (3) FPG level ≥ 100 mg/dL and/or taking antidiabetic medications; and (4) HDL-c levels < 40 mg/dL in men and <50 mg/dL in women	Kidney cancer	Age, sex, smoking habits, drinking habits, physical activity, and estimated glomerular filtration rate level
Chung et al. [43]	South Korea	6.1	227,102 (106,650)	62	BMI < 25 kg/m^2^	The presence of ≥3 of the following components: (1) WC ≥ 80 cm; (2) elevated FBG levels, defined as FPG levels ≥ 100 mg/dL; (3) TG levels ≥ 150 mg/dL; (4) HDL-c levels < 50 mg/dL for women; and (5) elevated BP (systolic BP ≥ 130 mmHg or diastolic BP ≥ 85 mmHg)	Pancreas cancer	Age, sex, smoking status, alcohol intake, physical activity, income level, and levels of hemoglobin, creatinine, alanine aminotransferase, and total cholesterol
Cui et al. [8]	China	13.76	93,956 (18,790)	51.08	BMI < 28 kg/m^2^	Metabolic status was defined as the presence of any one of four components: (1) serum TG ≥ 150 mg/dL or drug treatment for elevated TG; (2) serum HDL-c < 50 mg/dL in women or <40 mg/dL in men or drug treatment; (3) systolic BP ≥ 130 mmHg or diastolic BP ≥ 85 mmHg or drug treatment for elevated BP; and (4) FBG ≥ 100 mg/dL or drug treatment for elevated FBG.	Gastrointestinal cancer (esophageal cancer, gastric cancer, liver cancer, biliary cancer, pancreatic cancer, and colorectal cancer)	Age, sex, educational level, drinking, smoking, physical exercise, family history of cancer, salt intake, highly sensitive C-reactive protein, and alanine transaminase
Dibaba et al. [36]	USA	14	43,546 (43,546)	NR	BMI < 25 kg/m^2^	Presence of at least 3 metabolic factors, including: (1) high WC > 88 cm for women, (2) dyslipidemia or self-reported history of elevated cholesterol level, (3) high BP or self-reported history of hypertension, and (4) self-reported history of diabetes	Breast cancer	Age, race, BMI, education, region, physical activity, smoking, marital status, family history of breast cancer, ovary status, hysterectomy, hormonal therapy use, and ovary status BMI interaction
Florio et al. [15]	North America		1,168,733 (708,468)	64	BMI < 25 kg/m^2^	WC and WHR above the 75th percentiles	Primary liver cancer	Age, ethnicity, sex, alcohol consumption, cigarette smoking and study
Friedenreich et al. [16]	Canada	NA	406 (406)	48 years and 61.9 years	BMI < 25 kg/m^2^	Presence of 3 of the following risk factors: WC > 88 cm, TG > 150 mg/dL, HDL-c < 50 mg/dL, treatment of previously diagnosed hypertension, and FBG > 100 mg/dL	Endometrial cancer	Age, age at menarche, number of pregnancies > 20 weeks of gestation, type of HRT
Gunter et al. [17]	USA	8.2	917 (917)	65	BMI < 25 kg/m^2^	Insulin sensitivity (HOMA-IR–based definition of metabolic health, or insulin-based definition of metabolic health)	Postmenopausal Breast cancer	Age, ethnicity, age at menarche and menopause, parity, first-degree relative with breast cancer, education, alcohol consumption, physical activity, which of the two Woman Health Initiative studies each subject was enrolled in and, among those who participated in the clinical trials, which specific clinical trial arm they were assigned to and whether they were a member of the placebo or treatment group
Han et al. [18]	South Korea	5.4	8,406,308 (8,406,308)	48	BMI < 25 kg/m^2^	Defined as 3 or more of the 5 diagnostic criteria: abdominal circumference ≥ 90 cm, serum TG ≥ 150 mg/dL, serum HDL-c < 40 mg/dL, BP ≥ 130/85 mmHg or taking antihypertensive medication, and finally, FBG ≥ 100 mg/dL or taking antidiabetic medication.	Bladder cancer	Age, smoking history, alcohol history, exercise history, and income level
Hashimoto et al. [19]	Japan	5.5	15,607 (7023)	45.5	BMI < 25 kg/m^2^	Presence of one or more of the following four metabolic factors: (FBG, TG, HDL-c and BP)	Gastric cancer	Age, sex, alcohol consumption, smoking and exercise
Iyengar et al. [20]	USA	16	3460 (3460)	63.6	BMI < 25 kg/m^2^	Body fat including: whole body fat mass, whole-body fat, trunk fat mass index, fat mass index, fat mass of trunk, fat mass of leg at 75th percentiles	Breast cancer postmenopausal	Age at enrollment, educational attainment, race/ethnicity, age at menarche, age at first full-term birth, parity, age at menopause, oral contraceptive use, use of combined estrogen and progesterone therapy, use of unopposed estrogen therapy, physical activity, alcohol intake, and smoking
Kabat et al. [32]	USA	10	5175 (5175)	66.8	BMI < 25 kg/m^2^	Defined as having equal to or greater than 3 of the 5 following criteria: WC > 88 cm, TG > 150 mg/dL, HDL-c < 50 mg/dL, glucose > 100 mg/dL and systolic/diastolic BP > 130/85 mmHg or treatment for hypertension	Colorectal cancer	Age, smoking status, pack-years of smoking, alcohol intake, physical activity, aspirin intake, dietary calcium intake, dietary folate intake, caloric intake, oral contraceptives, HRT, parous/nulliparous, family history of colorectal cancer in first-degree relative, education, ethnicity, allocation to the Observational study component or specific arm of clinical trials
Kim et al. [21]	South Korea	5.4	7,421,410 (0)	46.5	BMI < 25 kg/m^2^	Defined as the presence ≥ 3 components of the metabolic syndrome	Prostate cancer	Age, smoking, alcohol drinking, exercise, and income
Kliemann et al. [34]	Multicountry (Denmark, Italy, the Netherlands, Spain, and the United Kingdom. In France, Germany, and Greece)	NA	694 (694)	54.8	BMI < 25 kg/m^2^	Based on the distribution of C-peptide concentration amongst the control population (tertile cut-points: 2.96 ng/mL and 4.74 ng/mL), and were classified as MH if below the first tertile of C-peptide and MU if above	Endometrial cancer	Study center, fasting status, age at blood collection, time of day at blood collection, menopausal status, exogenous hormone use, phase of menstrual cycle at blood collection, age at menopause, age at menarche, parity, hormone use, physical activity index, smoking status, educational level, alcohol intake, height, energy intake, and diabetes
Kwon et al. [22]	South Korea	5.3	255,051 (109,683)	38	BMI = 18.5–22.9 kg/m^2^	At least 1 of the following metabolic abnormalities: (i) FBG ‡100 mg/dL or current use of glucose-lowering agents; (ii) BP ‡130/85mmHg or current use of BP-lowering agents; (iii) elevated TG level (>150 mg/dL) or current use of lipid-lowering agents; (iv) low HDL-c (<40 mg/dL in men or <50 mg/dL in women); or (v) insulin resistance, defined as a HOMA-IR score > 2.5	Thyroid cancer	NR
Liang et al. [24]	USA		5068 (5068)	66.7	BMI < 25 kg/m^2^	Defined as having 3 or more of the 5 following criteria: WC > 88 cm, TG > 150 mg/dL, HDL-c < 50 mg/dL, glucose > 100 mg/dL and systolic/diastolic BP > 130/85 mmHg or treatment for hypertension	Colorectal and colon cancer	Age, ethnicity, smoking, alcohol consumption, physical activity, total energy intake, dietary fiber, percent calories from fat, family history of colorectal cancer, non-steroid anti-inflammatory drugs use, and treatment arm in each clinical trial
Lin et al. [33]	Taiwan	13.7	5324 (1936)	44.1	BMI = 18.5–23.9 kg/m^2^	Presence of all the following conditions: FBG > 100 mg/dL; BP > 130/85 mmHg; fasting TG level > 150 mg/dL; HDL-c level < 40 mg/dL in men or <50 mg/dL in women	Cancer incidence overall	Sex, age, smoking status, alcohol use, regular exercise, marital status, education, average monthly income
Mahamat-Saleh et al. [42]	Multicountry (Denmark, Italy, the Netherlands, Spain, and the United Kingdom. In France, Germany, and Greece)	3	1740 (1740)	60.5	BMI < 25 kg/m^2^	Based on the distribution of C-peptide concentration amongst the control population (tertile cut-points: 2.96 ng/mL and 4.74 ng/mL), and were classified as MH if below the first tertile of C-peptide and MU if above the first tertile	Postmenopausal breast cancer	Age at blood collection, time of day at blood collection, fasting status at blood collection, age at menarche, age at first full-term pregnancy and parity, age at menarche, age at first full term pregnancy and parity, age at menopause, breastfeeding, ever use of contraceptive pills, ever use of menopausal hormonal therapy, physical activity index, alcohol consumption, smoking status, educational level, height, and energy intake
Moon et al. [41]	South Korea	8.7	2,668,255 (2,668,255)	55.85	BMI = 18.5–23 kg/m^2^	Presence of at least 3 of the 5 following criteria: (1) WC ≥ 88 cm, (2) TG ≥ 150 mg/dL, (3) HDL-c < 50 mg/dL, (4) glucose ≥ 100 mg/ dL, and (5) systolic/diastolic BP ≥ 130/85 mmHg or treatment for hypertension	Colorectal	Age, smoking, alcohol consumption, vigorous physical activity, moderate physical activity, walking, age at menarche, age at menopause, parity, breastfeeding, oral contraceptive use, and first-degree family history of cancer
Moore et al. [23]	USA	30	1528 (1010)	56	BMI < 25 kg/m^2^	Elevated non-FBG (>125 mg/dL)	Obesity-related cancers (postmenopausal breast cancer, female reproductive (i.e., cervical, endometrial, and uterine), colon, liver, gallbladder, pancreas, kidney, and esophageal adenocarcinoma)	Age, sex, height, education level, alcohol, cigarettes/day, and physical activity, BMI, WC, occurrence of elevated glucose and obesity during follow-up
Murphy et al. [25]	Multicountry (Denmark, Italy, the Netherlands, Spain, and the United Kingdom. In France, Germany, and Greece)	3.7	259 (119)	57.6	BMI < 25 kg/m^2^	Based on the distribution of C-peptide concentration amongst the control population (tertile cut-points: 2.96 ng/mL and 4.74 ng/mL), and were classified as MH if below the first tertile of C-peptide and MU if above the first tertile	Colorectal cancer, colon cancer, rectal cancer	Matching factors, height, smoking status, physical activity, education level, alcohol consumption, and dietary intakes of total energy, red and processed meats, and fibre
Nguyen et al. [40]	South Korea	4.9	107,332 (74,311)	57.8	BMI < 25 kg/m^2^	Participants with abnormalities in three of these indices were considered MU: (1) TG, (2) BP, (3) HDL-c, (4) WC, and (5) FBG	Thyroid cancer	Age, sex, smoking, alcohol consumption, physical activity, and education
Ogundiran et al. [26]	Nigeria	10	1208 (1208)	47	BMI < 25 kg/m^2^	Presence of WHR > 0.87, or WC > 82 cm	Breast cancer	Age at diagnosis or interview (categorical), ethnicity, education (categorical), age at menarche (continuous), number of live birth (categorical), age at first live birth (continuous), duration of breastfeeding (categorical), menopausal status, family history of breast cancer, benign breast disease, hormonal contraceptive use, alcohol drinking, and height (continuous)
Park et al. [27]	USA	6.4	16,619 (16,619)	60.2	BMI < 25 kg/m^2^	Metabolic abnormalities considered included: high WC (>88 cm); elevated BP (>130/85 mmHg or antihypertensive medication); previously diagnosed diabetes or antidiabetic drug treatment; and cholesterol-lowering medication use	Breast cancer	Age at baseline, race, education, age at menarche, breastfeeding history, age at first live birth, parity, HRT, oral contraceptive use, menopausal status at baseline, sister age at diagnosis of breast cancer, smoking history, alcohol consumption, and physical activity
Park et al. [38]	South Korea	7.2	6,713,278 (3,088,108)	54.6	BMI < 25 kg/m^2^	Defined as the presence of ≥3 of the following components: (1) WC ≥ 80 cm; (2) elevated FBG levels, defined as FPG levels ≥ 100 mg/dL; (3) TG levels ≥ 150 mg/dL; (4) HDL-c levels < 50 mg/dL for women; and (5) elevated BP (systolic BP ≥ 130 mmHg or diastolic BP ≥ 85 mmHg)	Thyroid cancer	Age, smoking status, alcohol consumption, physical activity, income, and chronic kidney disease
Park et al. [29]	South Korea	9	1,935,800 (1,935,800)	59.5	BMI < 25 kg/m^2^	Defined as the presence of ≥3 of the following components: (1) WC ≥ 80 cm; (2) elevated FBG levels, defined as FPG levels ≥100 mg/dL; (3) TG levels ≥ 150 mg/dL; (4) HDL-c levels < 50 mg/dL for women; and (5) elevated BP (systolic BP ≥ 130 mmHg or diastolic BP ≥ 85 mmHg)	Postmenopausal breast cancer	Age, age at menarche, age at menopause, hormone replacement therapy use after menopause, delivery, duration of breastfeeding, oral contraceptive use, family history of any cancer, drinking frequency per week during the last 1 year, smoking, and physical activity including vigorous physical activity, moderate physical activity, and walking per week
Park et al. [35]	South Korea	9	2,649,564 (2,649,564)	51.5	BMI < 23 kg/m^2^	Defined as the presence of ≥3 of the following components: (1) WC ≥ 80 cm; (2) elevated FBG levels, defined as FPG levels ≥ 100 mg/dL; (3) TG levels ≥ 150 mg/dL; (4) high HDL-c levels < 50 mg/dL for women; and (5) elevated BP (systolic BP ≥ 130 mmHg or diastolic BP ≥ 85 mmHg)	Endometrial cancer	Age, smoking, drinking, vigorous physical activity, moderate physical activity, walking, age at menarche, age at menopause, number of children, breast feeding, oral contraceptive use, and family history of cancer
Reeves et al. [37]	USA	14.4	7588 (7588)	71	BMI < 25 kg/m^2^	Having at least one of the following metabolic abnormalities: (1) elevated WC, (2) hypertension, and (3) diabetes	Postmenopausal breast cancer	Age, current hormone use, and family history of breast cancer
Shao et al. [39]	England, Wales and Scotland	9.1	1,475,692 (79,687)	56	BMI < 25 kg/m^2^	Participants who met 4 of the 6 criteria above were considered healthy: (i) systolic diastolic BP < 130/85 mmHg; (ii) C-reactive protein < 3 mg/L; (iii) triacylglycerols < 2.3 mmol/L; (iv) LDL-C < 3 mmol/L and no cholesterol-lowering medications; (v) HDL-c > 1 mmol/L; (vi) HbA1c < 42 mmol/mol and no diabetes medications	Lung cancer	Age, sex, education level, ethnicity, smoking status, smoking duration, family history of lung cancer, and personal history of emphysema/bronchitis
Shin et al. [28]	South Korea	9	185,743 (0)	NR	BMI = 18.5–23 kg/m^2^	Defined as ≥1 claim per year for the prescription of oral hyperglycemics or insulin medication, or a FBG ≥ 7 mmol/L (obtained from the health examination database). Hypertension was defined as the presence of ≥1 yearly claim for the prescription of an antihypertensive agent or systolic/diastolic BP ≥ 140/90 mmHg. Dyslipidemia was defined as the presence of ≥1 yearly claim for the prescription of an antihyperlipidemic agent or total cholesterol ≥ 6.21 mmol/L (obtained from the health examination database)	Colorectal cancer	Age and sex, smoking, drinking, exercise, and income
Sun et al. [30]	Multicountry (Sweden, Norway, Austria)	20	434,232 (247,495)	42.8	BMI < 25 kg/m^2^	Defined as the top tertile of the metabolic score. The metabolic score comprised midblood pressure, FPG, and TG	Rectal cancer, pancreatic cancer, renal cell cancer, liver, intrahepatic bile ducts, gallbladder cancer, other obesity-related cancers	Sex, baseline age, and smoking status and pack-years and stratified by cohort and date of birth
Winn et al. [31]	USA	9	6190 (3095)	NR	BMI < 25 kg/m^2^	Defined as the presence of ≥3 of the following components: hyperglycaemia (FBG ≥ 100 mg/dL), hypertension (diastolic BP ≥ 85 mmHg or systolic BP ≥ 130 mmHg), abdominal obesity (WC > 88 cm (female) or >102 cm (male)), elevated TG (≥150 mg/dL) and low HDL-c (<50 mg/dL (female) or <40 mg/dL (male)) or drug treatment for these parameters]	Breast, colorectal, uterine, ovarian, pancreatic, liver, gallbladder, kidney and thyroid cancer	Age, sex, race/ethnicity, education, household income, smoking status, alcohol use, daily hours sedentary, weekly physical activity, average daily caloric intake and survey year

Abbreviations: BIA, Biolectrical impedance analysis; BMI, body mass index; BP, blood pressure; FBG, fasting blood glucose; FPG, fasting plasma glucose; HDL-c, high-density lipoprotein cholesterol; HbA1c, glycated hemoglobin; HOMA-IR, homeostasis model assessment-estimated insulin resistance; HRT, hormone replacement therapy; LDL-C, low-density lipoprotein cholesterol; mmHg, millimeters of mercury; MH, metabolically healthy; MU, metabolically unhealthy; NR, not reported; TG, triglycerides; USA, United States of America; WC, waist circumference; WHR, waist-to-hip ratio.

## Data Availability

The data underlying this article are available in the article and in its online Supplementary Materials.

## Supplementary Materials

The following supporting information can be downloaded at: https://www.mdpi.com/article/10.3390/jcm15020538/s1, Supplementary material: PRISMA Checklist; Supplementary Material S1: Literature search strategy for all the databases; Supplementary Material S2: Excluded studies and reasons for exclusion; Supplementary Material S3: Results of the Joanna Briggs Institute (JBI) Appraisal Checklist for Cohort studies; Supplementary Material S4: Forest plot showing hazard ratios (HRs) for cancer risk in normal weight individuals with central adiposity vs. not central adiposity; Supplementary Material S5: Forest plot showing hazard ratios (HRs) for cancer risk in normal weight individuals with glucose metabolism abnormalities vs. without glucose metabolism abnormalities; Supplementary Material S6: Forest plot showing hazard ratios (HRs) for cancer risk in normal weight individuals with elevated cholesterol vs. not elevated cholesterol; Supplementary Material S7: Forest plot showing hazard ratios (HRs) for cancer risk in normal weight individuals with hypertension vs. without hypertension, and elevated triglycerides vs. not elevated triglycerides; Supplementary Material S8: Meta-regression analysis of (a) length of follow-up and risk of having cancer in normal-weight individuals with metabolic abnormalities; and (b) age and risk of having cancer in normal-weight individuals with metabolic abnormalities. The solid line indicates a linear relationship. The size of each data point is proportional to its statistical weight; Supplementary Material S9: Publication bias. Supplementary Material S10: PRISMA Checklist. Refs. [55,56,57] are listed in the Supplementary.

## Abbreviations

The following abbreviations are used in this manuscript:

BMI	Body mass index
CI	Confidence interval
DNA	Deoxyribonucleic acid
HDL	High-density lipoprotein
HOMA-IR	Homeostatic Model Assessment for Insulin Resistance
HR	Hazard ratio
JBI	Joanna Briggs Institute
LDL	Low-density lipoprotein
LFK	Luis Furuya Kanamori
MUNW	Metabolically unhealthy normal-weight
PRISMA	Preferred Reporting Items for Systematic Reviews and Meta-Analyses
