# Supplementary material for: Metabolic Syndrome Components and Cancer Risk in Normal-Weight Subjects: Systematic Review and Meta-Analysis in over 18 Million Individuals
Source: J Clin Med. 2026 Jan 9;15(2):538. doi: 10.3390/jcm15020538 (PMC12842133; doi:10.3390/jcm15020538)

Metabolic red flags and cancer risk in normal-weight subjects:

Systematic Review and Meta-analysis in over 18 million individuals

Contents

Supplementary material S1. Literature search strategy for all the databases 2

Supplementary material S2. Excluded studies and reasons for exclusion. 3

Supplementary material S3. Results of the Joanna Briggs Institute (JBI) Appraisal Checklist for Cohort studies. 4

Supplementary material S4. Forest plot showing hazard ratios (HRs) for cancer risk in normal weight individuals with central adiposity vs not central adiposity. 6

Supplementary material S5. Forest plot showing hazard ratios (HRs) for cancer risk in normal weight individuals with glucose metabolism abnormalities vs without glucose metabolism abnormalities. 7

Supplementary material S6. Forest plot showing hazard ratios (HRs) for cancer risk in normal weight individuals with elevated cholesterol vs not elevated cholesterol. 8

Supplementary material S7. Forest plot showing hazard ratios (HRs) for cancer risk in normal weight individuals with hypertension vs without hypertension, and elevated triglycerides vs not elevated triglycerides. 9

Supplementary material S8. Meta-regression analysis of a) length of follow-up and risk of having cancer in normal-weight individuals with metabolic abnormalities; and b) age and risk of having cancer in normal-weight individuals with metabolic abnormalities. The solid line indicates a linear relationship. The size of each data point is proportional to its statistical weight. 10

Supplementary material S9. Publication bias. 11

This supplementary material has been provided by the authors to give readers additional information about their work.

Supplementary material S1. Literature search strategy for all the databases

PubMed:<br>((((((((((((((("metabolic syndrome") OR ("metabolic abnormalities")) OR ("metabolically unhealthy")) OR ("metabolically obese")) OR ("central adiposity")) OR ("central obesity")) OR ("waist circumference")) OR ("waist-to-hip-ratio")) OR (hyperglycaemia)) OR (cholesterol)) OR (triglycerides)) OR ("high blood pressure")) OR ("hypertension")) OR ("metabolically abnormal")) OR ("insulin resistance")) AND (("normal-weight") OR ("body mass index"))) AND (((cancer) OR (neoplasm)) OR (tumor))<br>Filters: Observational Study	277 results
Embase:<br>#1: 'metabolic syndrome x' OR 'metabolic abnormalities' OR 'normal weight obesity' OR 'metabolically obese' OR 'central adiposity' OR 'waist circumference' OR 'waist hip ratio' OR 'hyperglycaemia' OR 'cholesterol' OR 'hypertriglyceridemia' OR 'hypertension' OR 'insulin resistance'<br>#2: 'normal weight' OR 'body mass index'<br>#3: 'cancer risk' OR 'malignant neoplasm' OR cancer OR 'neoplasm'<br>#4: 'prospective study' OR 'retrospective study' OR 'case control study' OR 'follow up'<br>#1 AND #2 AND #3 AND #4 AND 'longitudinal study'/de	220 results
Web of Science:<br>normal-weight metabolically unhealthy cancer (Topic)	72 results

Supplementary material S2. Excluded studies and reasons for exclusion.

1. Barberio, A. M., Alareeki, A., Viner, B., Pader, J., Vena, J. E., Arora, P., ... & Brenner, D. R. (2019). Central body fatness is a stronger predictor of cancer risk than overall body size. Nature communications, 10(1), 383.

Reason for exclusion: No information on the outcome of interest

2. Pasqual, E., O'Brien, K., Rinaldi, S., Sandler, D. P., & Kitahara, C. M. (2023). Obesity, obesity-related metabolic conditions, and risk of thyroid cancer in women: results from a prospective cohort study (Sister Study). The Lancet Regional Health–Americas, 23.

Reason for exclusion: Inappropriate population

Supplementary material S3. Results of the Joanna Briggs Institute (JBI) Appraisal Checklist for Cohort studies.

JBI Appraisal Checklist for Cohort studies												
Study	1	2	3	4	5	6	7	8	9	10	11	Total
Ärnlöv et al. (2010)	Y	Y	Y	Y	Y	Y	Y	Y	Y	N	Y	10/11
Arthur et al. (2020)	Y	Y	Y	Y	Y	Y	Y	Y	Y	N	Y	10/11
Cao et al. (2020)	Y	Y	Y	Y	Y	Y	Y	Y	Y	N	Y	10/11
Cho et al. (2020)	Y	Y	Y	Y	Y	Y	Y	Y	Y	N	Y	10/11
Cho et al. (2022)	Y	Y	Y	Y	Y	Y	Y	Y	Y	N	Y	10/11
Chung et al. (2011)	Y	Y	Y	Y	Y	Y	Y	Y	Y	N	Y	10/11
Cui et al. (2024)	Y	Y	Y	Y	Y	Y	Y	Y	Y	N	Y	10/11
Dibaba et al. (2018)	Y	Y	Y	Y	Y	Y	Y	Y	Y	N	Y	10/11
Florio et al. (2019)	Y	Y	Y	Y	Y	Y	Y	Y	Y	N	Y	10/11
Friedenreich et al. (2011)	N	Y	Y	Y	Y	Y	Y	Y	Y	N	Y	9/11
Gunter et al. (2015)	Y	Y	Y	Y	Y	Y	Y	Y	Y	N	Y	10/11
Han et al. (2021)	Y	Y	Y	Y	Y	Y	Y	Y	Y	N	Y	10/11
Hashimoto et al. (2020)	Y	Y	Y	Y	Y	Y	Y	Y	Y	N	Y	10/11
Iyengar et al. (2018)	Y	Y	Y	Y	Y	Y	Y	Y	Y	N	Y	10/11
Kabat et al. (2018)	Y	Y	Y	Y	Y	Y	Y	Y	Y	N	Y	10/11
Kim et al. (2019)	Y	Y	Y	Y	Y	Y	Y	Y	Y	N	Y	10/11
Kliemann et al. (2022)	N	Y	Y	Y	Y	Y	Y	Y	Y	N	Y	9/11
Kwon et al. (2019)	Y	Y	Y	N	N	Y	Y	Y	Y	N	Y	8/11
Liang et al. (2017)	Y	Y	Y	Y	Y	Y	Y	Y	Y	N	Y	10/11
Lin et al. (2021)	Y	Y	Y	Y	Y	Y	Y	Y	Y	N	Y	10/11
Mahamat-Saleh et al. (2023)	N	Y	Y	Y	Y	Y	Y	Y	Y	N	Y	9/11
Moon et al. (2022)	Y	Y	Y	Y	Y	Y	Y	Y	Y	N	Y	10/11
Moore et al. (2013)	Y	Y	Y	Y	Y	Y	Y	Y	Y	N	Y	10/11
Murphy et al. (2016)	N	Y	Y	Y	Y	Y	Y	Y	Y	N	Y	9/11

Nguyen et al. (2021)	Y	Y	Y	Y	Y	Y	Y	Y	Y	N	Y	10/11
Ogundiran et al. (2012)	N	Y	Y	Y	Y	Y	Y	Y	Y	N	Y	9/11
Park et al. (2017)	Y	Y	Y	Y	Y	Y	Y	Y	Y	N	Y	10/11
Park et al. (2020)	Y	Y	Y	Y	Y	Y	Y	Y	Y	N	Y	10/11
Park et al. (2021)	Y	Y	Y	Y	Y	Y	Y	Y	Y	N	Y	10/11
Park et al. (2022)	Y	Y	Y	Y	Y	Y	Y	Y	Y	N	Y	10/11
Reeves et al. (2012)	Y	Y	Y	Y	Y	Y	Y	Y	Y	N	Y	10/11
Shao et al. (2022)	Y	Y	Y	Y	Y	Y	Y	Y	Y	N	Y	10/11
Shin et al. (2017)	Y	Y	Y	Y	Y	Y	Y	Y	Y	N	Y	10/11
Sun et al. (2023)	Y	Y	Y	Y	Y	Y	Y	Y	Y	N	Y	10/11
Winn et al. (2023)	Y	Y	Y	Y	Y	Y	Y	Y	Y	N	Y	10/11

Abbreviations: N, no; NA, not applicable; NR, not reported; Y, yes.
Items of the Quality Assessment Tool for Observational Cohort and Cross-Sectional Studies:
1. Were the two groups similar and recruited from the same population?
2. Were the exposures measured similarly to assign people to both exposed and unexposed groups?
3. Was the exposure measured in a valid and reliable way?
4. Were confounding factors identified?
5. Were strategies to deal with confounding factors stated?
6. Were the groups/participants free of the outcome at the start of the study (or at the moment of exposure)?
7. Were the outcomes measured in a valid and reliable way?
8. Was the follow up time reported and sufficient to be long enough for outcomes to occur?
9. Was follow up complete, and if not, were the reasons to loss to follow up described and explored?
10. Were strategies to address incomplete follow up utilized?
11. Was appropriate statistical analysis used?

Supplementary material S4. Forest plot showing hazard ratios (HRs) for cancer risk in normal weight individuals with central adiposity vs not central adiposity.

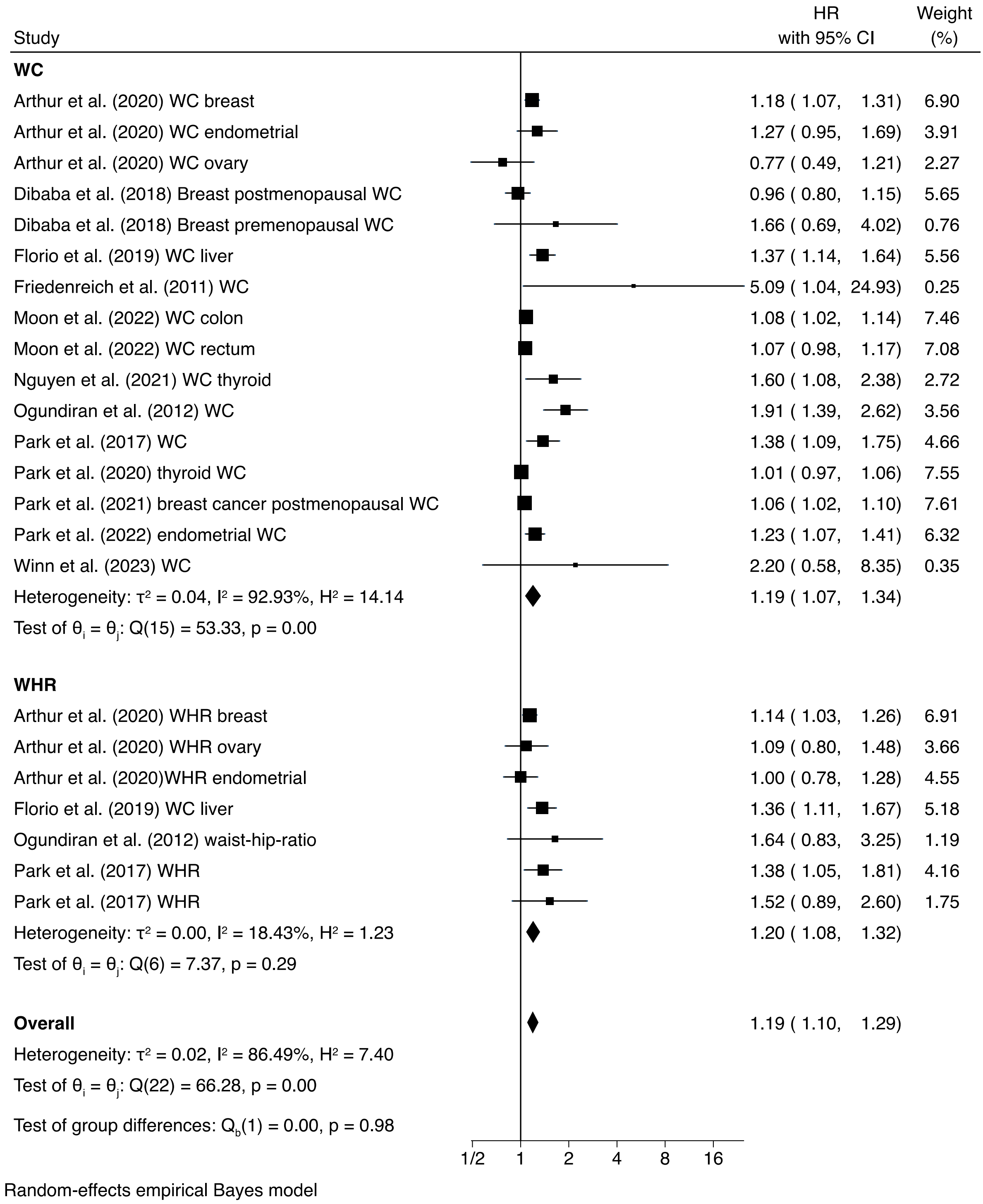

Supplementary material S5. Forest plot showing hazard ratios (HRs) for cancer risk in normal weight individuals with glucose metabolism abnormalities vs without glucose metabolism abnormalities.

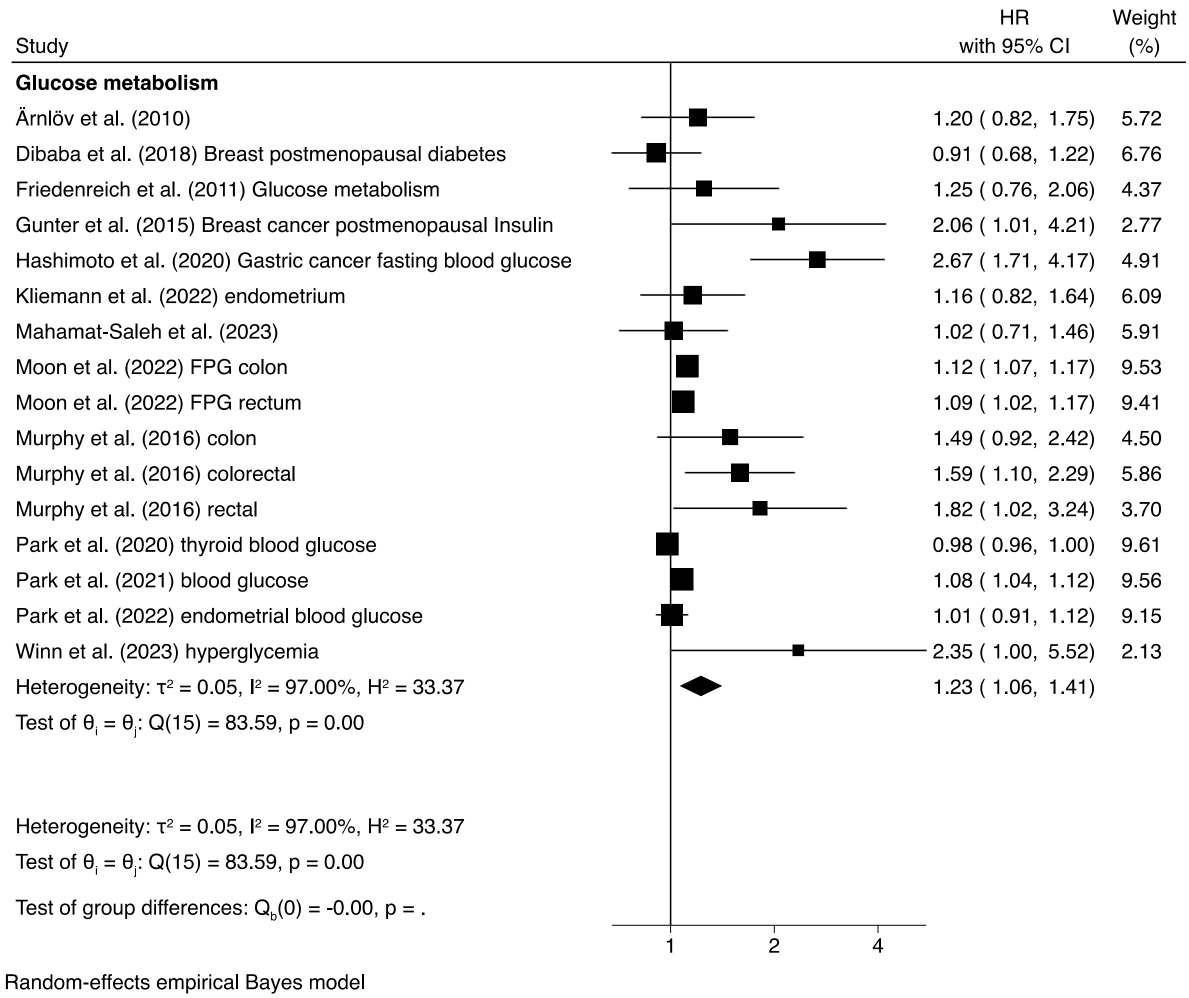

Supplementary material S6. Forest plot showing hazard ratios (HRs) for cancer risk in normal weight individuals with elevated cholesterol vs not elevated cholesterol.

Study	HR with 95% CI	Weight (%)
**Cholesterol**		
Dibaba et al. (2018) Breast postmenopausal cholesterol	0.99 (0.60, 1.64)	0.92
Dibaba et al. (2018) Breast postmenopausal cholesterol	1.14 (1.05, 1.24)	12.96
Hashimoto et al. (2020) Gastric cancer cholesterol	1.41 (0.86, 2.30)	0.97
Moon et al. (2022) HDL colon	1.03 (0.98, 1.08)	17.10
Moon et al. (2022) HDL rectum	1.02 (0.95, 1.09)	14.67
Nguyen et al. (2021) cholesterol thyroid	1.32 (1.03, 1.70)	3.27
Park et al. (2020) thyroid cholesterol	1.15 (1.13, 1.18)	19.71
Park et al. (2021) dyslipidemia	1.00 (0.97, 1.04)	18.56
Park et al. (2022) Endometrial cholesterol	1.05 (0.96, 1.15)	11.70
Winn et al. (2023) cholesterol	0.61 (0.17, 2.20)	0.15
Heterogeneity: $\tau^2 = 0.00$, $I^2 = 76.37\%$, $H^2 = 4.23$	1.07 (1.02, 1.13)	
Test of $\theta_i = \theta_j$: Q(9) = 61.17, p = 0.00		
Heterogeneity: $\tau^2 = 0.00$, $I^2 = 76.37\%$, $H^2 = 4.23$		
Test of $\theta_i = \theta_j$: Q(9) = 61.17, p = 0.00		
Test of group differences: $Q_b(0) = 0.00$, p = .		

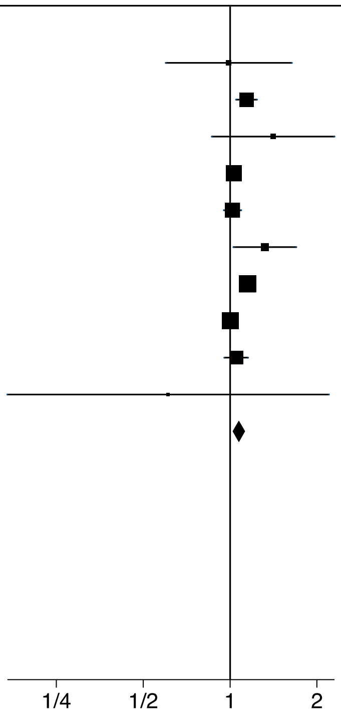

Random-effects empirical Bayes model

Supplementary material S7. Forest plot showing hazard ratios (HRs) for cancer risk in normal weight individuals with hypertension vs without hypertension, and elevated triglycerides vs not elevated triglycerides.

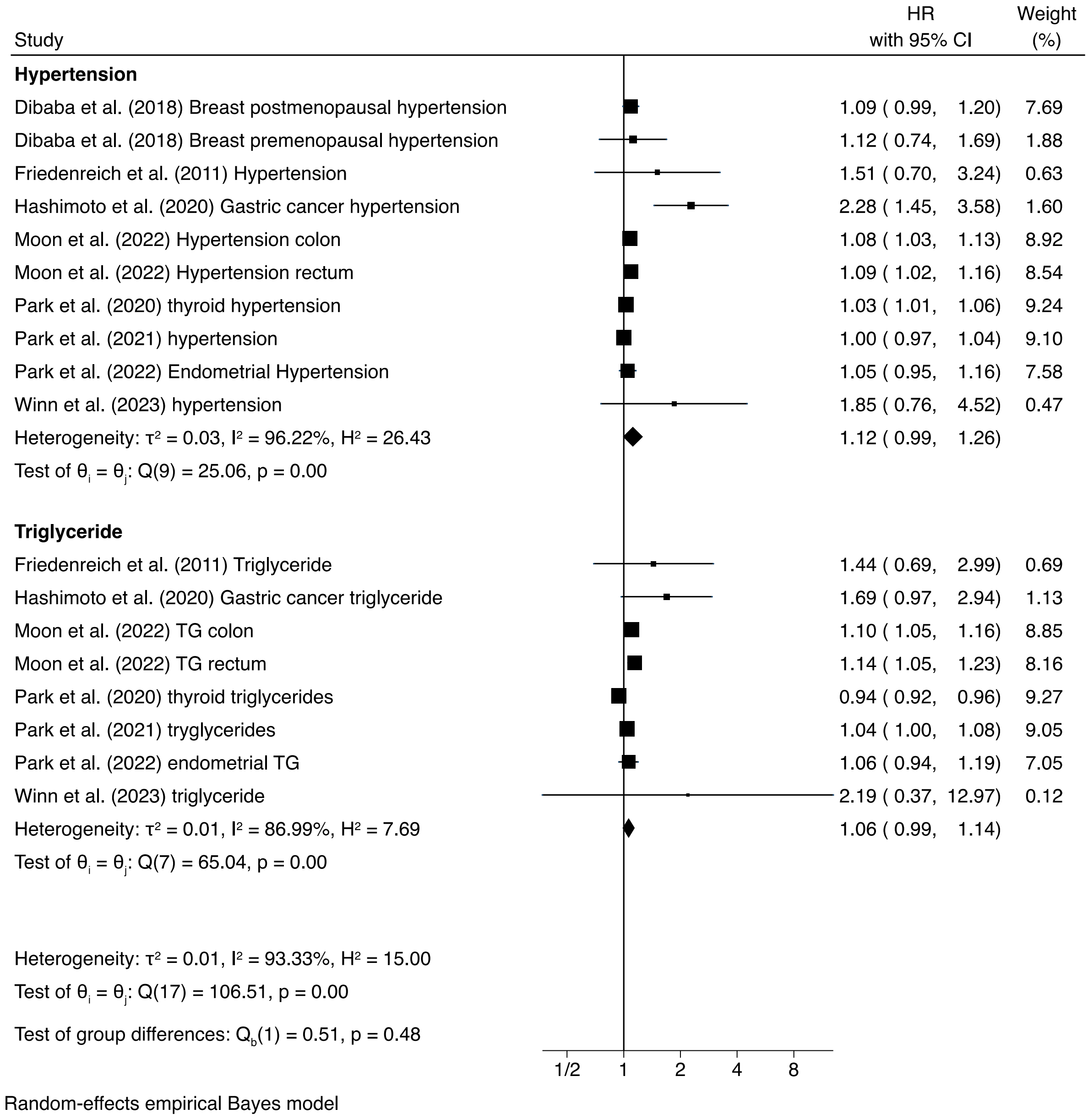

Supplementary material S8. Meta-regression analysis of a) length of follow-up and risk of having cancer in normal-weight individuals with metabolic abnormalities; and b) age and risk of having cancer in normal-weight individuals with metabolic abnormalities. The solid line indicates a linear relationship. The size of each data point is proportional to its statistical weight.

a)

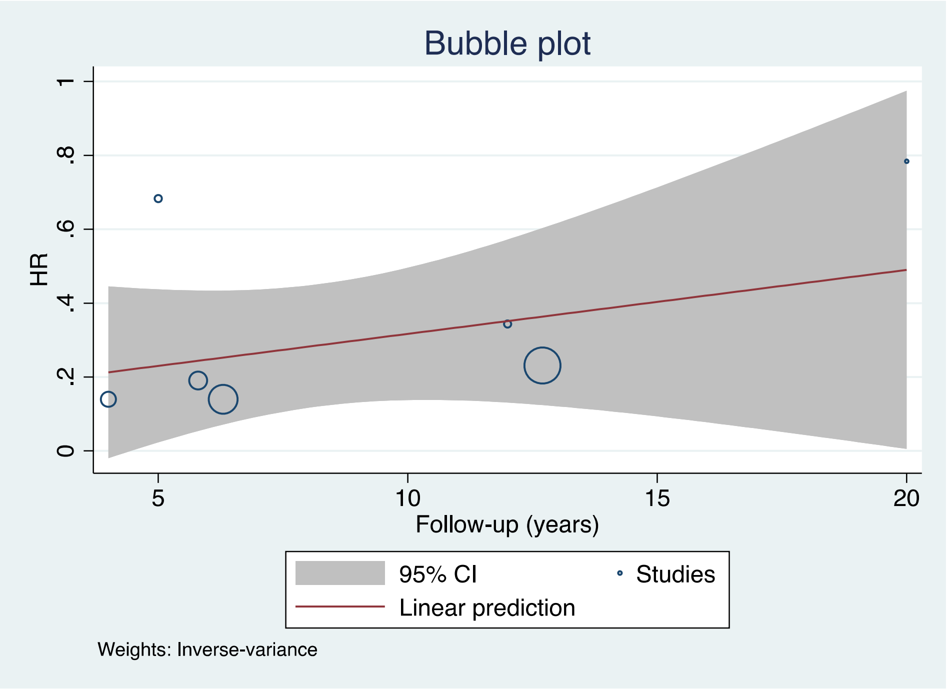

b)

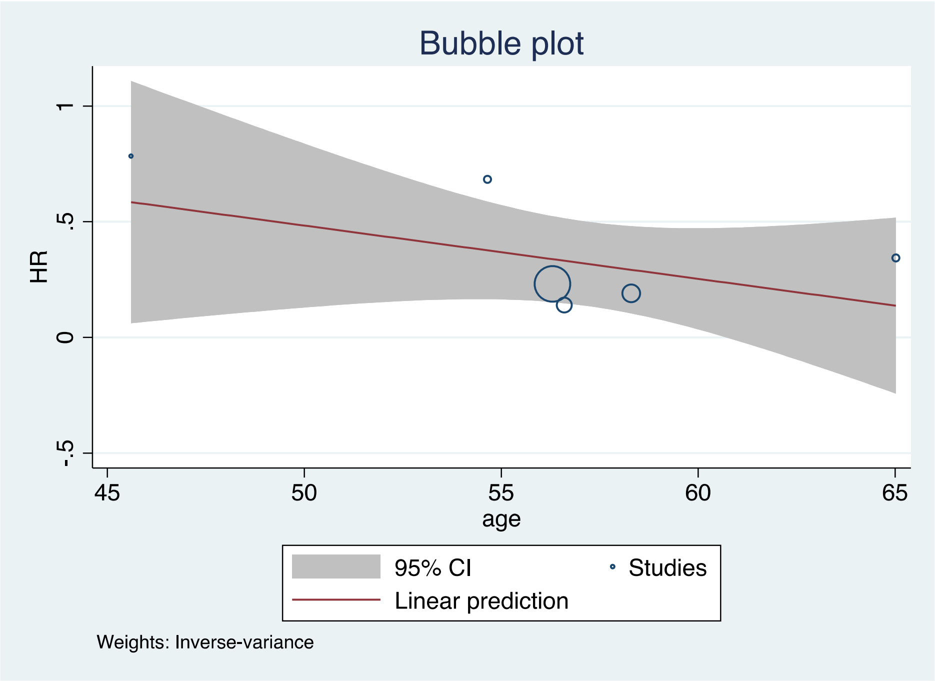

Supplementary material S9. Publication bias.

A) Doi-plot of metabolic syndrome and risk of cancer.

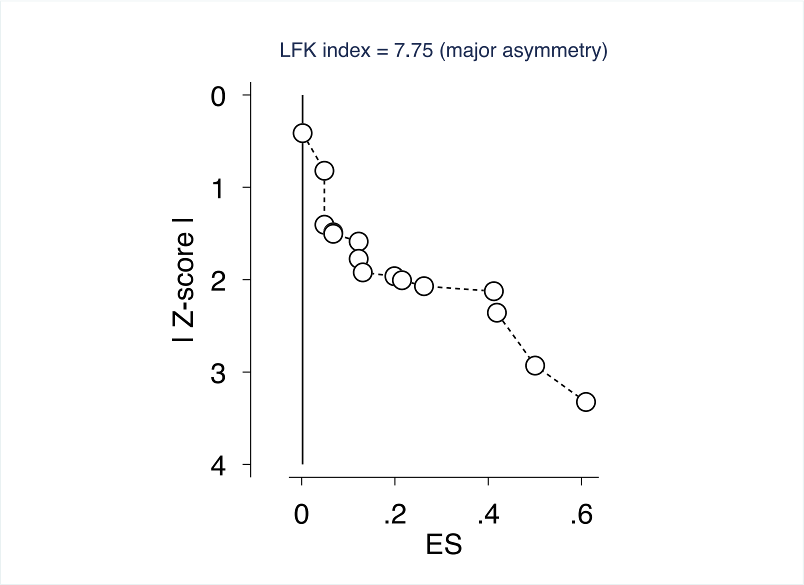

B) Doi-plot of central adiposity and risk of cancer.

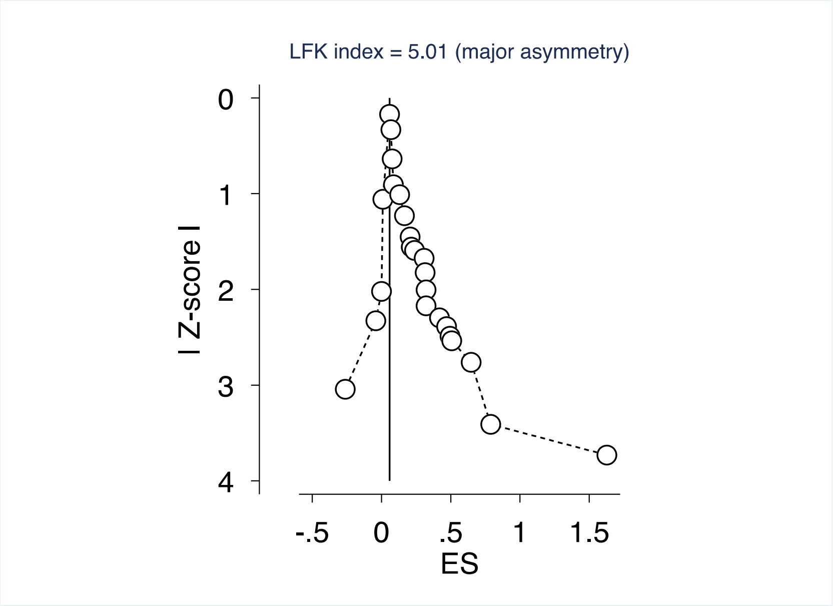

C) Doi-plot of abnormal glucose metabolism and risk of cancer.

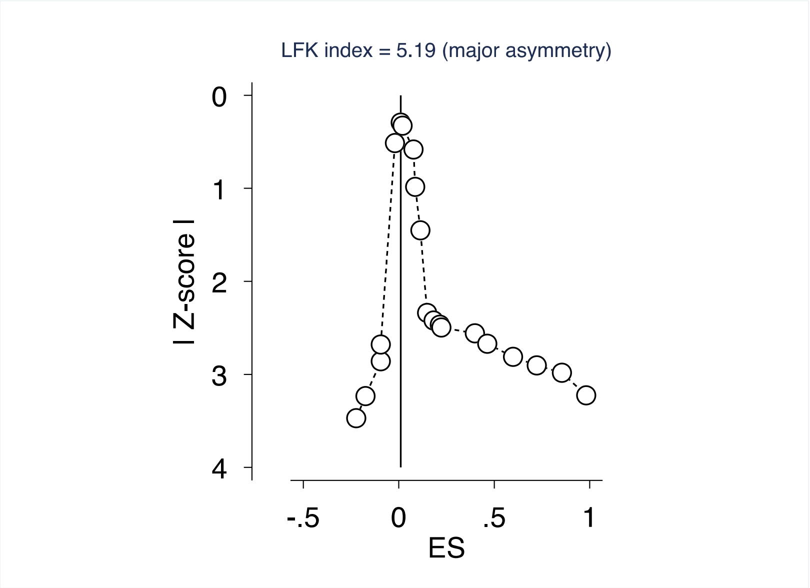

D) Doi-plot of elevated cholesterol and risk of cancer.

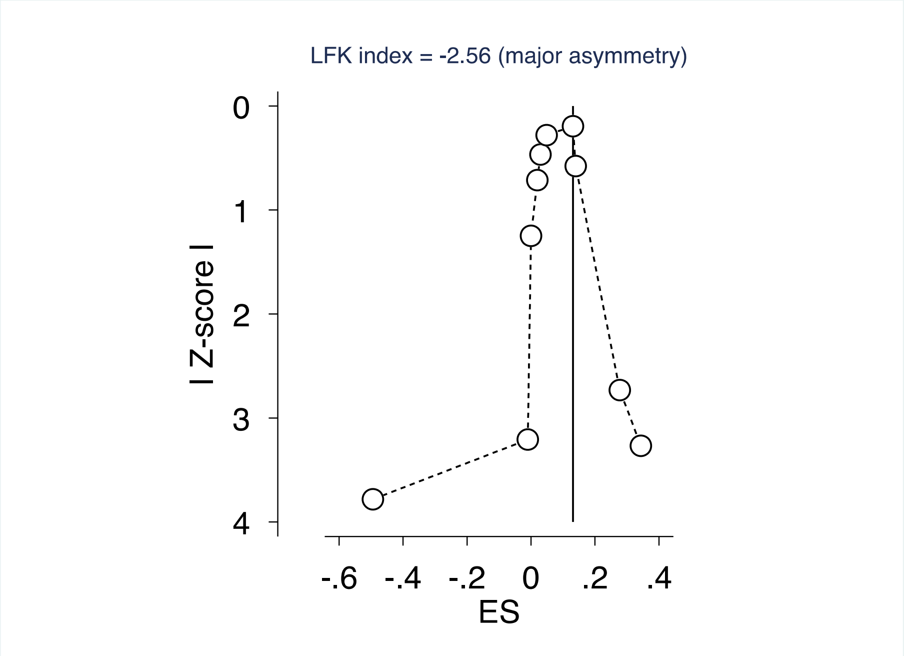

E) Doi-plot of hypertension and risk of cancer.

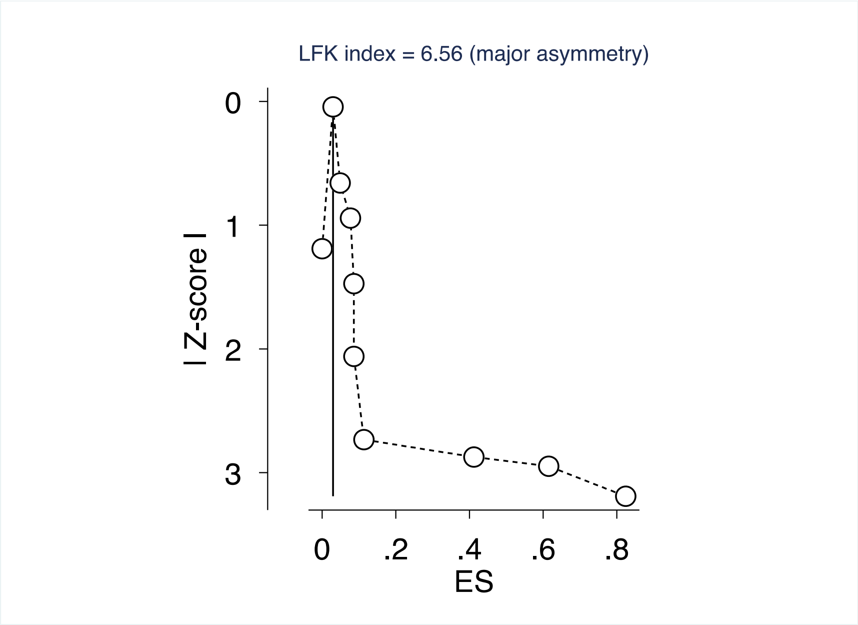

F) Doi-plot of elevated triglycerides and risk of cancer.

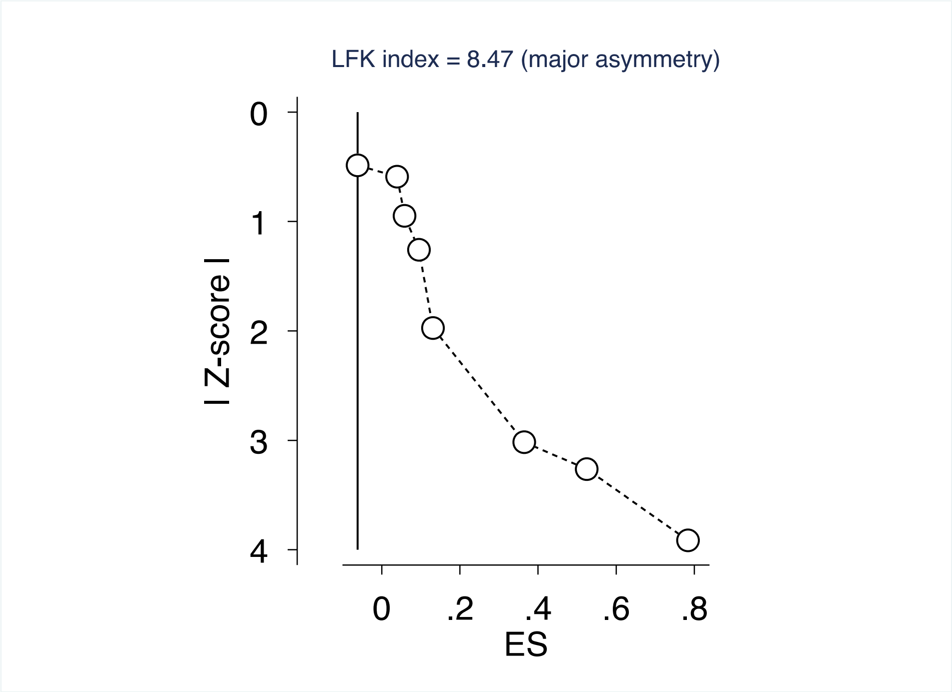

Supplement: Supplementary file 1 [file jcm-15-00538-s001.zip › Supplementary material S1-S9.pdf]
